# Design and rationale for an open-label, randomized, controlled pilot trial to evaluate the changes in blood uremic toxins in patients with chronic kidney disease by dietary therapy with sake lees

**DOI:** 10.1007/s10157-023-02450-x

**Published:** 2024-02-10

**Authors:** Toshiaki Tokumaru, Tadashi Toyama, Yusuke Nakade, Hisayuki Ogura, Megumi Oshima, Shiori Nakagawa, Motoe Furuichi, Shinji Kitajima, Norihiko Sakai, Miho Shimizu, Yasunori Iwata, Takashi Wada

**Affiliations:** 1 Department of Nephrology and Rheumatology, Kanazawa University, Takara-Machi 13-1, Kanazawa City, Ishikawa, 920-8641 Japan; 2 Department of Nutrition Management, Kanazawa University Hospital, Kanazawa, Japan; 3 Innovative Clinical Research Center, Kanazawa University, Kanazawa, Japan; 4 Division of Infection Control, Kanazawa University Hospital, Kanazawa, Japan

**Keywords:** Sake lees, Chronic kidney disease, Dysbiosis, Uremic toxins, D-amino acids

## Abstract

**Background:**

Patients with chronic kidney disease (CKD) reportedly show dysbiosis, which is the imbalance of gut microbiome. Dysbiosis increases the uremic toxin level in the intestine, and uremic toxins transfer into the blood, causing CKD progression. Sake lees, a traditional Japanese fermented food, may help reduce uremic toxins by altering the gut microbiome. Additionally, D-alanine, which is present in sake lees, may have a renoprotective effect. The present pilot study aims to evaluate the effect of adding sake lees to the standard CKD dietary therapy in reducing blood uremic toxins.

**Methods:**

This pilot study is a single-center, open-label, randomized controlled trial. Twenty-four patients with CKD will be enrolled and allocated 1:1 to the intervention and control groups. The intervention group will receive standard CKD dietary therapy with an additional intake of 50 g of sake lees per day for 8 weeks, whereas the control group will only receive standard CKD dietary therapy. The primary endpoint is the change in serum indoxyl sulfate after 8 weeks. The secondary endpoint is the plasma D-alanine and fecal microbiome changes.

**Conclusion:**

This pilot study provides insight into the development of a new diet focused on gut microbiome and D-amino acids in patients with CKD.

**Clinical trial registration:**

This protocol was approved by the Clinical Trial Review Board of Kanazawa University Hospital on October 27, 2022 (2022-001 [6139]) and available to the public on the website of the Japan Registry of Clinical Trials on November 22, 2022 (jRCT1040220095).

**Supplementary Information:**

The online version contains supplementary material available at 10.1007/s10157-023-02450-x.

## Introduction

Patients with chronic kidney disease (CKD) reportedly show dysbiosis, which is the imbalance of gut microbiome [1]. Dysbiosis increases the uremic toxin levels in the intestine, and uremic toxins transfer into the blood, causing CKD progression [2]. Recently, CKD treatment focusing on the gut microbiome has been performed in clinical trial [3]. Probiotics and prebiotics are considered the primary treatment candidates for dysbiosis. For example, a type of probiotics or prebiotics can alter the gut microbiome in CKD patients and can reduce the blood uremic toxins or blood urea nitrogen levels [4, 5].

Sake lees is a fermented food commonly consumed in Japan [6]. It is generated in the fermentation process of Japanese sake (rice wine). Sake lees contains bacteria and dietary fiber and improves the gut microbiome of patients with chronic constipation [7]. Additionally, sake lees also contains D-alanine and D-serine, which are mainly produced by bacteria [8, 9] and could potentially have renoprotective effects [8, 9].

Based on these backgrounds, sake lees may have renoprotective effects by improving the gut microbiome and producing D-alanine and D-serine by the bacteria present in sake lees. To evaluate the renoprotective effects, it is necessary to monitor the blood uremic toxin levels, which may be altered by sake lees intake and accelerate CKD progression.

This pilot study aims to evaluate whether the addition of sake lees to the standard CKD dietary therapy decreases the blood uremic toxin levels as compared to the standard CKD dietary therapy only.

## Materials and methods

### Study setting

**Fig. 1:**
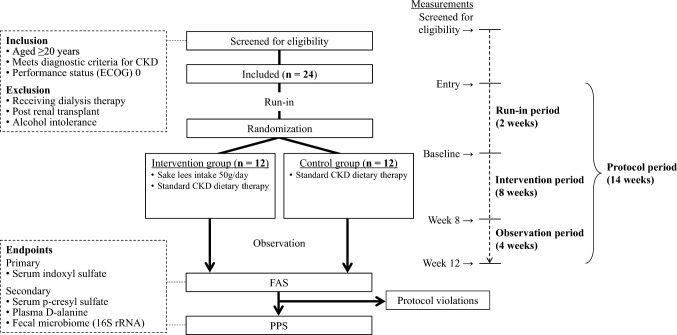
The study flowchart. *CKD* chronic kidney disease, *ECOG* Eastern Cooperative Oncology Group, *FAS* full analysis set, *PPS* per protocol set

This pilot study is a single-center, open-label, randomized controlled trial with a target sample size of 24 participants. Figure 1 shows a summary of the trial. The main eligibility criterion is meeting the diagnostic criteria for CKD. After a 2-week run-in period, participants will be allocated 1:1 to the intervention and control groups. During the intervention period, the intervention group will receive standard CKD dietary therapy with an additional intake of 50 g of sake lees per day for 8 weeks, whereas the control group will only receive the standard CKD dietary therapy. After the intervention, both groups will be observed for a period of 4 weeks. The primary endpoint is the change in serum indoxyl sulfate. The secondary endpoints are the changes in the serum p-cresyl sulfate and plasma D-alanine levels as well as fecal microbiome. The measurements for the endpoints will be performed at baseline and at 8 and 12 weeks.

### Eligibility criteria

The inclusion criteria are as follows: age ≥20 years, estimated glomerular filtration rate (eGFR): 15 to <60 mL/min/1.73 m^2^, meeting the diagnostic criteria for CKD [10], performance status score (Eastern Cooperative Oncology Group [11]) of 0, and regular visits at the hospital. The exclusion criteria are as follows: on dialysis therapy, post-renal transplant, cancer-bearing, body mass index of <16 kg/m^2^, immunocompromised, inflammatory bowel disease, liver dysfunction, daily use of antibiotics, history of antimicrobial therapy within the past 4 weeks of study entry, daily intake of sake lees, alcohol intolerance, history of alcohol dependence, and physician’s determination of unfitness for this study.

### Intervention and control

The intervention group will receive the standard CKD dietary therapy with an additional intake of 50 g of sake lees per day for 8 weeks. The standard CKD dietary therapy consists of energy 30–35 kcal/kg-standard body weight/day, protein 0.6–1.0 g/kg-standard body weight/day, and salt <6 g/day, according to the guidelines for CKD dietary therapy [12]. The control group will not be adjusted for the addition of energy or protein to the intervention group. Potassium and phosphorus should be restricted as needed. The control group will only receive the standard CKD dietary therapy for 8 weeks and is prohibited from taking sake lees. Both groups are prohibited from consuming functional foods or drugs labeled to improve the intestinal environment during the protocol period. The consumption of yogurt or other common fermented foods is acceptable. During the protocol period, the additions or dosage changes of drugs originally taken are allowed. The use of antimicrobials or intestinal agents during the protocol period is a protocol violation.

### Intervention details

The sake lees that will be used in this study are made from a Japanese sake called “Kakuma no Sato” and are purchased from the manufacturer in Japan. According to the nutritional information on the product label, the main nutrients per 100 g of sake lees are as follows: energy: 209 kcal, protein: 7.2 g, fat: 0.6 g, carbohydrates: 30.8 g, salt: 0 g, dietary fiber: 5.2 g, and alcohol: 8 g. All participants will use the same lot of sake lees. We will prepare a sake lees recipe to make it easy for the participants to cook sake lees. The participants will also be allowed to cook sake lees as they liked. The sake lees amount per meal and the mealtime will be left to the participants. The participants will record their sake lees intake daily on a designated recording form. The recording form will also include the frequency of bowel movements, form of stool, and use of laxatives. Nutritional guidance by registered dietitian will be provided at included, baseline, and 8 and 12 weeks.

### Endpoints

The primary endpoint of this study is the level of blood indoxyl sulfate, a metabolite of intestinal origin, and a surrogate marker of renal function [13]. Given that this study hypothesized changes in the levels of intestinal uremic toxins due to sake lees intake, the primary endpoint is the change in serum indoxyl sulfate concentration at baseline and at 8 weeks. Table 1 shows the list of secondary endpoints. The secondary endpoints include the changes in serum p-cresyl sulfate and plasma D-alanine levels and fecal microbiome. Changes in eGFR and proteinuria will not be considered endpoints in this study, because the intervention period of 8 weeks is too short to expect significant changes in those parameters. The measurement schedule for each outcome variable is shown in Table 2. Basic data, such as serum creatinine and potassium levels, will also be assessed for eligibility verification or safety purposes.

**Table 1:** Primary and secondary endpoints

	Sample	Endpoints
Primary	Blood	Serum indoxyl sulfate
Secondary	Blood	Serum p-cresyl sulfate
		Plasma D-alanine
		Plasma D-serine
	Feces	Indole
		P-cresol
		Acetic acid
		Propionic acid
		Butyric acid
		pH
		Microbiome (16S rRNA gene sequence analysis)
	Urine	Urinary protein
		pH
		Estimated salt excretion
		MCP-1
		KIM-1
	Questionnaire	CSS
		PAC-QOL
		FFQg

*MCP-1* monocyte chemotactic protein 1, *KIM-1* kidney injury molecule 1, *CSS* constipation scoring system, *PAC-QOL* patient assessment of constipation quality-of-life questionnaire, *FFQg* food frequency questionnaire based on food groups

**Table 2:** Measurements schedule

	Screened for eligibility	Entry	Baseline	Week 8	Week 12
Check the criteria	✓				
Basic data	✓		✓	✓	✓
Medical questionnaire		✓			
Recording form			✓	✓	✓
*Outcomes*					
Blood					
Serum indoxyl sulfate			✓	✓	✓
Serum p-cresyl sulfate			✓	✓	✓
Plasma D-alanine			✓	✓	✓
Plasma D-serine			✓	✓	✓
Feces					
Indole			✓	✓	✓
P-cresol			✓	✓	✓
Acetic acid			✓	✓	✓
Propionic acid			✓	✓	✓
Butyric acid			✓	✓	✓
pH			✓	✓	✓
Microbiome			✓	✓	✓
Urine					
Urinary protein			✓	✓	✓
pH			✓	✓	✓
Estimated salt excretion			✓	✓	✓
MCP-1			✓	✓	✓
KIM-1			✓	✓	✓
Questionnaire					
CSS			✓	✓	✓
PAC-QOL			✓	✓	✓
FFQg		✓		✓	

*MCP-1* monocyte chemotactic protein 1, *KIM-1* kidney injury molecule 1, *CSS* constipation scoring system, *PAC-QOL* patient assessment of constipation quality-of-life questionnaire, *FFQg* food frequency questionnaire based on food groups

### Sample size

The sample sizes for the intervention and control groups will be 12 cases each. The number of cases is set based on feasibility and the recommended number of cases for the pilot study [14]. The maximum sample size allowed for entry will be 30 in total for both groups, considering the possibility of discontinuations.

### Recruitment, run-in, and randomization

Recruitment will be conducted at the Department of Nephrology and Rheumatology, Kanazawa University Hospital (Kanazawa City, Ishikawa, Japan). The patients who met the eligibility criteria will be given a 2-week run-in period to eliminate the influence of food or drugs on the intestinal environment. During the run-in period, the intake of sake lees is prohibited. The participants are prohibited from consuming functional foods or drugs labeled to improve the intestinal environment. The consumption of yogurt or other common fermented foods is acceptable. A dynamic allocation strategy using the minimization method will be used for randomization. The allocation ratio is set at 1:1, with allocation adjustments for age (<75 or ≥75 years) and eGFR (15 to <30 or 30 to <60 mL/min/1.73 m^2^). Subjects will be automatically randomized using a web-based case registration and randomization system.

### Data and sample collection

Data will be collected at baseline and at 8 and 12 weeks. In addition to the primary and secondary endpoints, basic data, such as age, sex, blood pressure, height, body weight, skeletal muscle mass, body fat mass, red blood cell count, hemoglobin, hematocrit, and aspartate aminotransferase, alanine aminotransferase, blood urea nitrogen, serum creatinine, serum uric acid, serum potassium, serum phosphorus, serum albumin, and total cholesterol levels, will be collected. Participants’ bowel movements will be assessed using a constipation scoring system [15] and a patient-rated constipation quality-of-life questionnaire [16]. At baseline and week 8, data on nutritional intake (energy, protein, salt, and dietary fiber) will be collected using a food frequency questionnaire based on food groups [17]. The participants will record the amount of their sake lees intake and compliance status of prohibited food or drugs during the protocol period. This record will be collected by the research group on week 12.

Specimens will be collected at baseline and 8 and 12 weeks. The plasma and serum will be centrifuged and stored at −80 °C. Participants will take a defined meal for dinner the day before the blood sampling and the morning of the blood sampling. Detailed information regarding the defined meal is provided in **Supplementary Fig. 1**. The feces for indole and p-cresol analysis are sampled by participants in a stool container using a specimen-collection spoon. The stool container will be collected and stored at −80 °C. The feces for gut microbiome analysis will be sampled using a brush kit (TechnoSuruga Laboratory Co., Ltd., Shizuoka, Japan) and stored refrigerated. Spot urine will be sampled at the time of the visit and centrifuged, and the supernatant will be stored at −80 °C.

### Statistical analysis

The primary and secondary endpoints will be tested using unpaired *t* tests for the measurement changes from baseline to week 8 between the intervention and control groups. As a secondary analysis, the changes between the intervention and control groups at baseline, 8 and 12 weeks will be tested using repeated measures analysis of variance. A multivariable regression analysis will be performed on the change in serum indoxyl sulfate levels from baseline to week 8, using age, sex, and eGFR at baseline as covariates. A two-tailed significance level of *p* < 0.05 will be used.

## Discussion

This pilot study involving CKD patients will evaluate the changes in blood uremic toxin levels during standard CKD dietary therapy with sake lees intake. The intervention group will receive 50 g per day of sake lees for 8 weeks in addition to the standard CKD dietary therapy, and the changes in serum indoxyl sulfate levels will be compared with that of the control group.

Spherical carbon adsorbents have been used as a therapeutic agent to target uremic toxins in the intestine of CKD patients. In a large clinical trial in CKD patients [carbonaceous oral adsorbent’s effectiveness on progression of chronic kidney disease (CAP-KD study)], patients receiving spherical carbon adsorbents had a significantly slower decrease in eGFR [18]. Similarly, in the multinational, randomized, double-blind, placebo-controlled evaluating prevention of progression in CKD (EPPIC) and the carbonaceous oral adsorbent’s effectiveness on progression of chronic kidney disease (KAP-KD) trials, the decrease in eGFR was significantly slower in patients receiving spherical charcoal [19, 20]. However, the adherence to the oral administration of spherical carbon adsorbents was poor due to the high treatment doses and oral discomfort. Constipation as a side effect also contributes to poor adherence.

A common dietary therapy for dysbiosis includes intake of probiotics such as yogurt and prebiotics such as fruits and vegetables. However, CKD patients may have difficulty actively consuming yogurt due to phosphorus restriction and fruits or vegetables due to potassium restriction. Dietary supplements have also been tested as probiotics and prebiotics for CKD patients. For example, probiotics and prebiotics have been reported to reduce the p-cresyl sulfate levels in CKD patients [21]. These supplements are also difficult to obtain and relatively expensive; hence, making a generalization may be difficult.

Sake lees is affordable and easily available in Japan. Sake lees contains dietary fiber and many of bacteria types. It is also low in phosphorus and potassium and contains no salt, making it suitable for CKD patients. As one of its effects, a study on people with constipation reported that consumption of a drink containing sake lees for 4 weeks changed the occupancy rate of intestinal bacteria [22]. Constipation also improved when 50 g of sake lees was consumed for 3 weeks [7].

In addition to the bacteria and dietary fiber, sake lees contains D-alanine and D-serine, which may potentially have renoprotective effects [8, 9]. We have also confirmed that D-alanine is produced by bacteria present in the body [23]. Therefore, consumption of sake lees may exert a renoprotective effect due to the D-amino acids and D-amino acid-producing bacteria present in sake lees.

Based on these findings, the intake of sake lees in CKD patients has a renoprotective effect. However, the dietary therapy using sake lees for CKD patients has not been studied. Before examining the effectiveness of sake lees intake in CKD patients, the effect of sake lees on the uremic toxin levels or gut microbiome needs to be explored. Additionally, the changes in D-alanine and D-serine levels in the body should be confirmed.

Our study protocol was approved by the Clinical Trial Review Board of Kanazawa University Hospital on October 27, 2022 (2022-001 [6139]) and available to the public on the website of the Japan Registry of Clinical Trials on November 22, 2022 (jRCT1040220095). We enrolled the first patient on December 21, 2022. By the end of August 2023, we enrolled 19 patients and further recruitment is still ongoing. This pilot study will end in March 2024. Based on the results obtained from this pilot study, a study design will be developed for a future randomized controlled trial to examine the renoprotective effects of sake lees.

## Conclusion

This pilot study provides insight into the development of a new diet focused on gut microbiome and D-amino acids in patients with CKD. Based on the results of our pilot study, a study design will be developed for a future randomized controlled trial to examine the renoprotective effects of sake lees intake.

### Supplementary Information

Below is the link to the electronic supplementary material.

Supplementary file1 (PPTX 63 KB)
