# Supplementary material for: Design and rationale for an open-label, randomized, controlled pilot trial to evaluate the changes in blood uremic toxins in patients with chronic kidney disease by dietary therapy with sake lees
Source: Clin Exp Nephrol. 2024 Feb 10;28(5):440–6. doi: 10.1007/s10157-023-02450-x (PMC11033224; doi:10.1007/s10157-023-02450-x)
Supplement: Supplementary file 1 — Supplementary file1 (PPTX 63 KB) [file 10157_2023_2450_MOESM1_ESM.pptx]

## Slide 1
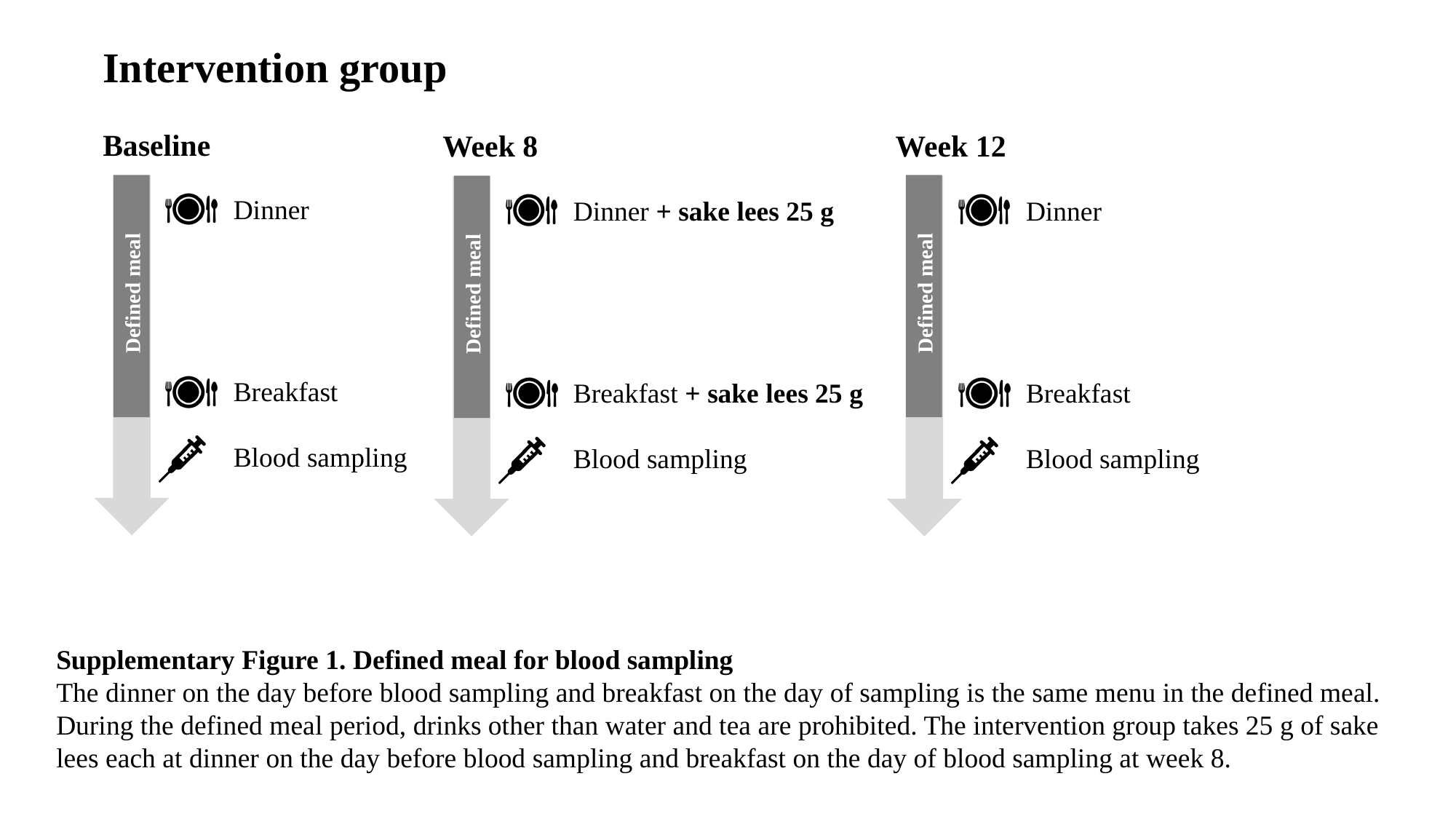

Intervention group
Baseline
Week 8
Week 12
Dinner
Breakfast
Blood sampling
Dinner + sake lees 25 g
Breakfast + sake lees 25 g
Blood sampling
Dinner
Breakfast
Blood sampling
 Defined meal
 Defined meal
 Defined meal
Supplementary Figure 1. Defined meal for blood sampling
The dinner on the day before blood sampling and breakfast on the day of sampling is the same menu in the defined meal. During the defined meal period, drinks other than water and tea are prohibited. The intervention group takes 25 g of sake lees each at dinner on the day before blood sampling and breakfast on the day of blood sampling at week 8.

## Slide 2
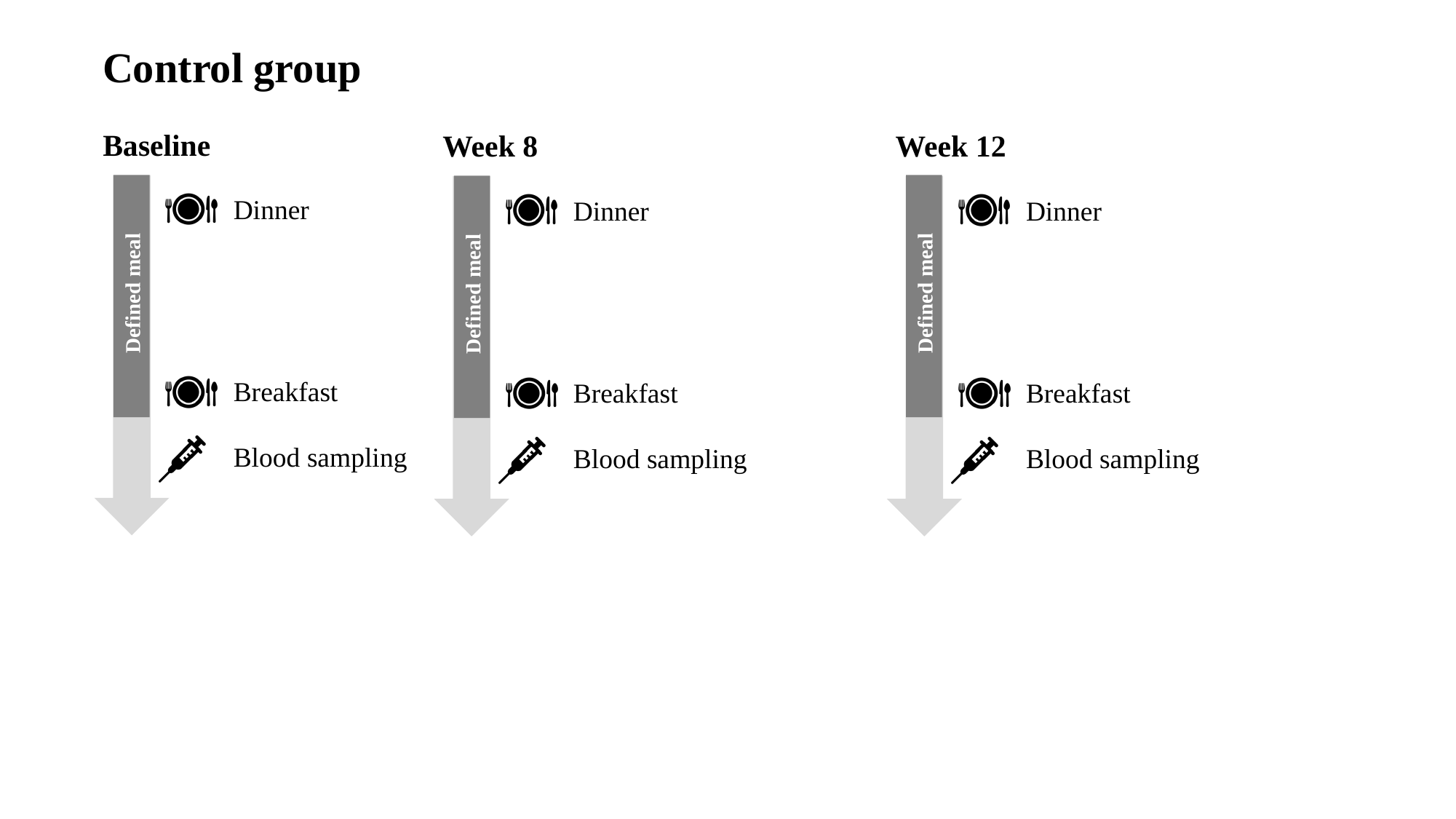

Control group
Baseline
Week 8
Week 12
Dinner
Breakfast
Blood sampling
Dinner
Breakfast
Blood sampling
Dinner
Breakfast
Blood sampling
 Defined meal
 Defined meal
 Defined meal
